# Distinct landscape and clinical implications of therapy-related clonal hematopoiesis

**DOI:** 10.1172/JCI180069

**Published:** 2024-10-01

**Authors:** Koichi Takahashi, Daisuke Nakada, Margaret Goodell

**Affiliations:** 1 Departments of Leukemia and Genomic Medicine, The University of Texas MD Anderson Cancer Center, Houston, Texas, USA.; 2 Department of Molecular and Human Genetics and; 3 Department of Molecular and Cellular Biology, Baylor College of Medicine, Houston, Texas, USA.

## Abstract

Therapy-related clonal hematopoiesis (t-CH) is defined as clonal hematopoiesis detected in individuals previously treated with chemotherapy and/or radiation therapy. With the increased use of genetic analysis in oncological care, the detection of t-CH among cancer patients is becoming increasingly common. t-CH arises through the selective bottleneck imposed by chemotherapies and potentially through direct mutagenesis from chemotherapies, resulting in a distinct mutational landscape enriched with mutations in DNA damage-response pathway genes such as *TP53*, *PPM1D*, and *CHEK2*. Emerging evidence sheds light on the mechanisms of t-CH development and potential strategies to mitigate its emergence. Due to its unique characteristics that predominantly affect cancer patients, t-CH has clinical implications distinct from those of CH in the general population. This Review discusses the potential mechanisms of t-CH development, its mutational landscape, mutant-drug relationships, and its clinical significance. We highlight the distinct nature of t-CH and call for intensified research in this field.

## Introduction

Clonal hematopoiesis (CH) is characterized by the preferential expansion of hematopoietic stem cells (HSCs) that possess somatic driver mutations (1). Historically, most CH research has concentrated on the general population. However, with the rapid integration of genomic sequencing into the field of oncology, there has been a marked increase in the identification of CH instances among cancer patients (2). This uptick is partially explained by the more frequent application of genomic diagnostics in oncological settings as opposed to the general health screenings of the broader population — a discrepancy that may diminish as genomic assays become more commonplace in preventive medicine and consumer genetics (e.g., 23andMe) (3).

Investigations into CH within oncological cohorts have yielded a consistent observation: cancer patients exhibit a mutational bias toward genes that are part of the DNA-damage response (DDR) pathway, including but not limited to *TP53*, *PPM1D*, and *CHEK2* (4, 5). Mutations in these genes confer a degree of chemoresistance to HSCs, resulting in a clonal advantage of cells with the mutations when treated with DNA-damaging chemotherapeutic agents (6–8). This suggests that the enrichment of DDR pathway mutations observed in the posttreatment cancer patient cohort reflects clonal selection processes induced by chemotherapy. Indeed, a number of studies using murine model systems have demonstrated that these chemotherapy treatments facilitate selective expansion of DDR-mutated HSCs (6, 8–10). These findings illuminate the mechanisms of clonal selection and resistance that arise as an adaptive response to the genotoxic stress of cancer therapy.

This Review aims to summarize the landscape of therapy-related CH (t-CH), highlighting the distinct mutational profiles that differentiate it from CH observed in the general population. Our objective is to dissect the contributing role of therapeutic interventions in the evolution of CH and to consider the broader implications for the prognosis and management of patients with t-CH.

## Mutational landscape of t-CH

The t-CH concept lacks a universally accepted definition. Still, it is commonly applied to describe the clonal expansion of HSCs harboring somatic mutations that is observed in patients who have undergone chemotherapy, a demographic predominantly composed of cancer patients (4, 11). The seminal discovery of CH in cancer patients arose incidentally through the Cancer Genome Atlas (TCGA) studies, which utilized blood samples as germline controls during the genomic sequencing of cancer tissues (12). Unexpectedly, these “control” samples revealed the presence of mutations associated with myeloid malignancies, such as *DNMT3A*, *TET2*, *ASXL1*, and *JAK2* mutations, prompting a reevaluation of these blood samples as reservoirs of somatic mutations rather than pristine controls. Although these findings were not necessarily indicative of t-CH, as TCGA subjects were primarily untreated for cancer at the time of sampling, they provided an initial framework for understanding CH in a cancer-affected population.

Subsequent, more focused investigations at Memorial Sloan Kettering Cancer Center (MSKCC) leveraged the MSK-IMPACT platform, analyzing matched normal blood samples to identify CH in 8,810 individuals with a history of cancer therapy (4). This study found CH in 25% of the cancer patients. Notably, the most common CH mutations (e.g., *DNMT3A* and *TET2*) were well represented, but distinct variants had marked enrichment. Mutations were skewed toward *TP53*, *PPM1D*, *ATM*, and *CHEK2*, diverging from the mutational patterns typically seen in the general population. These findings were further corroborated by Bolton et al. and Stonestrom et al., who each examined an expanded MSK-IMPACT cohort of 24,146 and 42,714 patients with cancer (including 8,810 patients studied by Coombs, et al.; ref. 4), confirming that approximately 30% of them carried CH (5, 13). Internal comparison between patients previously treated with chemotherapy and those without treatment has found a significant enrichment of *PPM1D*, *TP53*, and *CHEK2* mutations in patients with prior treatments (5).

Although smaller in scale, several ancillary studies have provided additional granularity to the mutational spectrum of t-CH, exploring its manifestation across various cancer subtypes and therapeutic contexts (detailed in Table 1) (14–50). These studies consistently validate the initial findings, suggesting a mutational convergence within t-CH marked by the enrichment of mutations in DDR pathway genes. One of the notable patterns observed across these studies is the significant enrichment of *PPM1D* mutations in patients with lymphoma and myeloma undergoing autologous stem cell transplantation. This finding may reflect the unique therapeutic exposures in these patient populations or the potential enrichment of these mutations in mobilized stem cell products. Furthermore, a consistent observation across these studies is the higher-than-expected prevalence of CH in cancer patients compared with the general population. However, it remains unclear whether this increased prevalence reflects the impact of prior therapy or whether cancer patients inherently have a higher propensity for developing CH. This is partially due to the fundamental challenges in CH studies, including variability in sequencing platforms, the sensitivity of detection, and the heterogeneity of patient exposures across different studies, which complicates interstudy comparisons.

## Factors distinguishing t-CH from de novo CH

**Figure 1:**
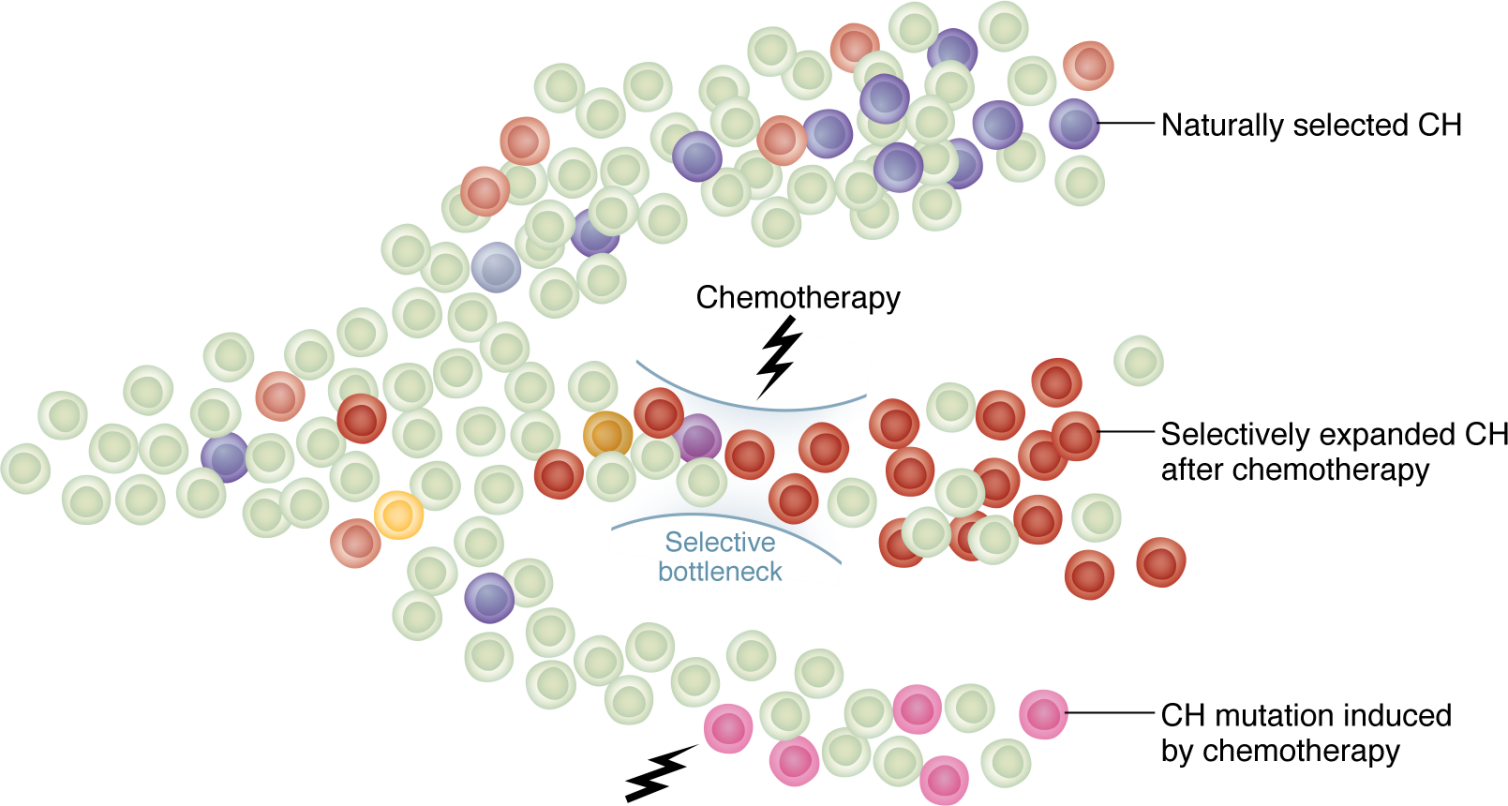
Conceptual summary of the mechanisms of t-CH development and dn-CH. The top branch depicts naturally occurring (de novo) CH. The middle branch depicts preexisting CH selected by the therapeutic pressure of chemotherapy, leading to t-CH. The bottom branch depicts t-CH directly caused by chemotherapy.

The distinct mutational landscapes between t-CH and de novo CH (dn-CH) invite a multitude of hypotheses. While the appearance of t-CH mutations after DNA-damaging chemotherapy may suggest that the mutations result from the treatments, deep sequencing of hematopoietic cells from individuals with cancer has revealed the presence of driver mutations, albeit at low levels, prior to chemotherapy (8, 51). Thus, the prevailing concept is that mutant clones generally exist before treatments, and chemotherapy exposure then selects for the expansion of these clones. Chemotherapy reduces the fitness of the “normal” hematopoietic stem and progenitor cells, while cells with DDR mutations are positively selected. This selective bottleneck enables the outgrowth of clones with specific mutations that confer a survival advantage (Figure 1). This phenomenon has been substantiated by diverse experimental models, including in vitro competitive culture experiments and in vivo chimeric transplant models, demonstrating that cytotoxic chemotherapy can promote the positive selection of mutant HSC clones. Table 2 compares this and other characteristics of t-CH with dn-CH.

Nonetheless, emerging evidence increasingly suggests that chemotherapy may directly induce CH mutations, particularly within pediatric populations (52, 53). In pediatric cancer patients, platinum chemotherapy has been shown to cause t-CH with driver mutations (41, 54) (Figure 1). Hagiwara et al. demonstrated that procarbazine treatment may induce *STAT3* mutations in T cells in pediatric cancer patients (55). Moreover, Bertrums et al. reported that thiopurines and platinum-based therapies are associated with therapy-induced somatic mutations in HSCs and therapy-related myeloid neoplasms (t-MNs) in pediatric cancer patients (56). In adult populations, Diamond et al. showed that platinum and melphalan chemotherapies led to treatment-induced mutations in t-MN samples (57). Recent studies have also revealed that treatment-induced somatic mutations are detectable in normal HSCs in adult populations treated with melphalan, platinum compounds, and other chemotherapeutic agents (58, 59). Although the proportion of therapy-induced versus therapy-expanded mutations remains unclear, it is plausible that a subset of t-CH arises from the mutagenic effects of chemotherapy. Notably, the mutational landscape of t-CH appears to differ between adult and pediatric populations: in adults, mutations selected through cancer therapy predominate, while therapy-induced mutations are more frequently observed in pediatric patients. This difference may be attributed to the preexisting pool of CH mutations in adults, which are less likely to be present in pediatric patients. Consequently, a systematic investigation is warranted to delineate the contributions of selective versus mutagenic mechanisms in the development of t-CH.

Beyond the direct effects of chemotherapy, inherent genetic predispositions contribute to the formation of distinct mutational profiles in t-CH. Patients with hereditary cancer syndromes who possess germline mutations that predispose them to malignancies could be more inclined to develop specific CH mutations. This predisposition potentially creates a distinct pool of CH mutations within the host, which then become subject to the selective pressures of chemotherapy, resulting in unique mutational profiles in t-CH. For instance, some studies have shown that patients with hereditary cancer predisposition syndromes (e.g., *BRCA* mutations or *TP53* mutations) have a higher risk of developing t-MNs, suggesting that these syndromes may predispose patients to an increased pool of CH mutations, thereby laying the groundwork for subsequent t-MN development (60, 61).

Environmental factors also contribute to the divergence of mutational spectra between t-CH and dn-CH. Cancer patients often have distinct exposure histories, such as increased rates of tobacco use or alcohol consumption, which are known to influence the mutation spectrum in HSCs (e.g., *ASXL1* mutation and tobacco smoking) (4, 5, 62, 63). These exposures can induce specific genetic alterations or promote clonal expansion of certain mutant clones, thereby modulating the mutational landscape of t-CH. Nonetheless, the predominant factor influencing the t-CH landscape remains chemotherapy treatment, with both germline predispositions and environmental factors likely contributing to the pool of CH mutations that are subsequently subjected to chemotherapy-induced selection.

## Mutant-specific implications of t-CH

**Figure 2:**
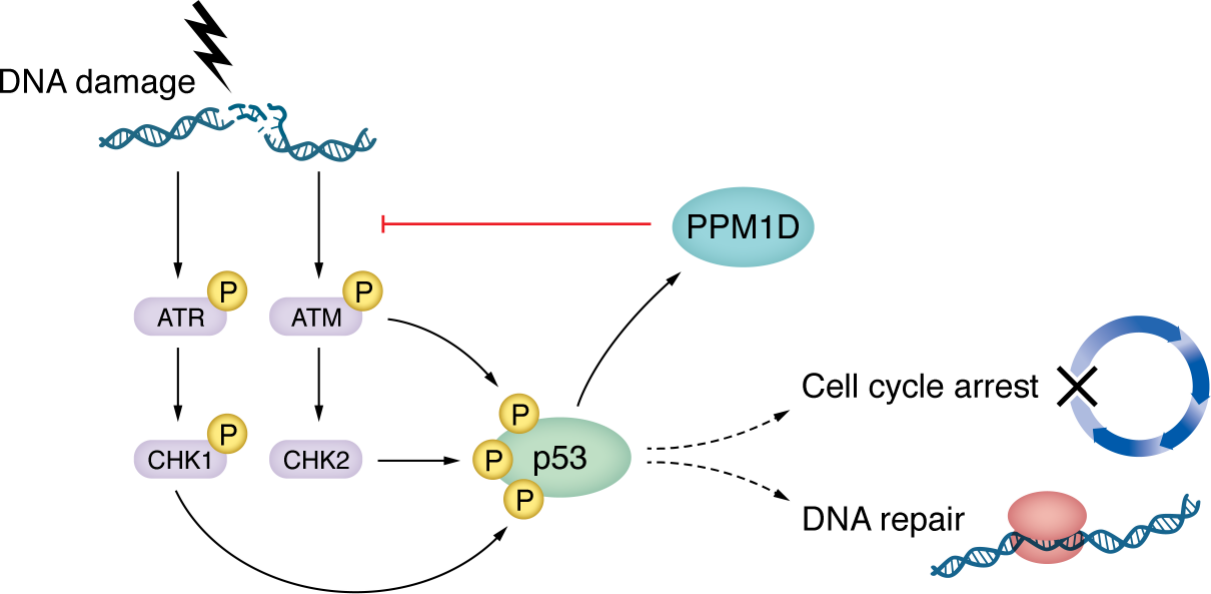
A simplified view of the DDR pathway depicts some of the genes that commonly carry mutations after exposure to DNA-damaging agents. ATM and ATR are sensors of DNA damage. p53 is a major transducer of the signal through its activity as a transcription factor regulating multiple downstream responses. PPM1D becomes transcriptionally upregulated by phosphorylated p53. PPM1D is a phosphatase; once produced, it dephosphorylates many of the events that have activated the DNA-damage cascade, serving to reduce the response (hence, PPM1D activating mutations found in t-CH partially phenocopy TP53 loss-of-function mutations).

Within the spectrum of t-CH, mutations in DDR pathway genes are notably predominant (Figure 2 and Table 3). Key players such as *TP53*, *PPM1D*, and *CHEK2* have been identified as recurrently mutated, signifying their potential role in the pathogenesis of t-CH.

### TP53 mutations.

Mutations in *TP53*, the gene encoding the tumor suppressor p53, are relatively infrequent in CH within the general population (64). However, they represent some of the most common aberrations in t-CH. Despite the ongoing debate regarding the prognostic impact of *TP53* mutations in the general CH context (65), their association with a high risk of leukemic transformation is widely acknowledged (66, 67).

The p53 protein is central to the DDR, integrating signals from various DNA-repair mechanisms. When DNA damage is sensed, p53 is phosphorylated by ATM or other mediators (68). This activation enables p53 to transcribe additional components of the DDR response, including p21, which suppresses the cell cycle to allow time for DNA repair or to induce apoptosis. Once the response is attenuated, many cells can resume division. However, when p53 function is abrogated by mutation, variant cells continue dividing and can outcompete other progenitors. Given the centrality of p53 to the DDR, it is unsurprising that reduced function leads to the expansion of cells with *TP53* mutations compared with wild-type counterparts. Accordingly, the enrichment of *TP53* mutations is observed following treatment with various chemotherapeutic agents.

For instance, using a chimeric mouse bone marrow–transplant model, Wong et al. demonstrated that treatment with ENU, an alkylating agent, selectively expands *Trp53* mutant cells over wild-type cells (8). Similarly, Bondar et al. showed that radiation treatment promotes selective expansion of *Trp53* mutant cells using a similar transplant model (69). Consistent with the data from mouse models, robust correlations have been observed in human cohorts between *TP53* mutations in t-CH and prior treatment with platinum-based drugs, alkylating agents, and ionizing radiation (5, 70).

In addition to these associations with classic cytotoxic chemotherapy, clonal expansion of *TP53*-mutated cells has also been observed with molecularly targeted agents, such as PARP inhibitors and lenalidomide (9, 15, 39). Recent observations have highlighted an increased incidence of *TP53* mutations in t-CH among ovarian and prostate cancer patients undergoing therapy with PARP inhibitors (15, 39). However, the underlying mechanisms remain to be elucidated.

Additionally, a significant association has been noted between *TP53* mutations in t-MNs and prior treatment with lenalidomide, an analog of thalidomide predominantly used to treat multiple myeloma (9). Thalidomide derivatives work in part by inducing the degradation of specific oncogenic proteins, such as IKAROS and CK1a, via these drugs’ target, CRBN (9, 71). Using mouse models with human *CRBN* sequence knocked in (as the mouse is inherently resistant to thalidomide analogs), Sperling et al. showed that lenalidomide treatment promoted the clonal dominance of *Trp53*-mutant cells over wild-type cells (9). Intriguingly, treatment with pomalidomide, another thalidomide analog, did not show the same degree of clonal selection. This difference is attributed to the extent of CK1a degradation: lenalidomide is a stronger degrader of CK1a compared with pomalidomide. CK1a degradation induces p53-dependent apoptosis in HSCs, causing cell death of wild-type HSCs, whereas *TP53*-mutant cells are resistant to lenalidomide-induced apoptosis. Conversely, pomalidomide is less toxic to wild-type HSCs and therefore does not efficiently select *TP53*-mutant cells. Given that pomalidomide is expected to provide similar treatment efficacy against multiple myeloma while reducing the positive selection for high-risk *TP53* clones, it is hypothesized that replacing lenalidomide with pomalidomide may decrease the risk of t-MN development in this context.

### PPM1D mutations.

*PPM1D* mutations rank as the fifth most common type in dn-CH. Strikingly, they are highly enriched in the context of t-CH (5, 64). The initial identification of *PPM1D* mutations in the blood of ovarian cancer patients first postulated them as biomarkers for ovarian cancer (72); however, further insights have clarified their role as markers of CH.

*PPM1D*, also known as WIP1, acts as a negative regulator of the p53 tumor suppressor, thereby playing a crucial role in the cellular response to DNA damage (73). Mutations in *PPM1D* tend to cluster in exon six at the carboxy terminal end of the protein, frequently resulting in truncations that prevent ubiquitination and subsequent degradation, leading to more stable expression of the PPM1D protein (6, 7). Such gain-of-function mutations partially phenocopy loss-of-function mutations in *TP53*, as both lead to a compromised p53-response pathway. Consistent with this, *TP53* and *PPM1D* mutations are often found together (coselected) in CH within the same individual, particularly in patients who have undergone extensive chemotherapy. However, when examined at the single-cell level, these mutations are mutually exclusive (74). This mutual exclusivity of DDR mutations in CH is further exemplified by the finding that carriers of germline DDR mutations, such as those in *ATM*, *CHEK2*, and *TP53*, are devoid of *PPM1D* somatic mutations (75). Instead, these germline mutation carriers were significantly enriched for somatic mutations in the remaining allele, leading to biallelic inactivation of *ATM* or *TP53*. While the significance of biallelic inactivation of DDR genes remains unclear, biallelic inactivation may be required to gain a competitive advantage among the pool of already competitive, heterozygously mutated cells.

From a clinical standpoint, HSCs with *PPM1D* mutations demonstrate a fitness advantage when exposed to cytotoxic chemotherapy, particularly agents that cause DNA damage, such as alkylating agents and platinum-based drugs (5, 6). The fitness of PPM1D-mutant HSCs was compared with that of *TP53*-mutated cells, where *TP53* mutations have been shown to confer a more potent selective advantage (10). Without selective pressure, *PPM1D*-mutant clones appear to have a very mild advantage over nonmutant counterparts. They cannot outcompete wild-type cells in mice over short time periods (6, 7), but may have an advantage over more extended periods (10), accounting for their prevalence in the general population and common CH clones.

Despite their prevalence in t-CH, the leukemic potential of *PPM1D* mutations remains uncertain. Animal models with *Ppm1d* truncation mutations do not consistently develop myeloid malignancies (6). In human cases of t-CH or t-MNs, the variant allele frequencies (VAFs) of *PPM1D* mutations are typically low, raising questions about their capacity to drive leukemogenesis (6, 76). However, detecting *PPM1D* mutations as t-CH in peripheral blood stem cells (PBSCs) was associated with an increased risk of t-MN development (77), suggesting a complex interplay between these mutations and leukemic transformation.

When additional oncogenic drivers were introduced into *PPM1D*-mutant murine bone marrow cells, frank malignancy developed, indicating that PPM1D does not negatively affect the cellular fitness of transformed cells (10). Nonetheless, hematologic malignancies with *PPM1D* mutations in the dominant clone are rare. It is possible that because *PPM1D*-mutant clones are typically found alongside *TP53*-mutant clones, which potently lead to transformation, the *PPM1D*-mutant clones may be outcompeted in generating malignancies. Notably, similar *PPM1D* mutations are commonly found in solid tumors, such as brain-stem gliomas, and have been associated with poor prognosis, supporting the concept that they are indeed compatible with frank malignancy (reviewed in ref. 78).

### CHEK2 mutations.

Mutations in *CHEK2*, a gene encoding a checkpoint kinase integral to DNA-damage signaling and cell cycle control, are observed with notable frequency in t-CH. This stands in contrast to their rarity within cases of dn-CH, suggesting a therapy-associated mechanism (4, 5, 79). To date, comprehensive functional characterization of *CHEK2* mutations in HSCs has yet to be conducted, leaving a gap in understanding the adaptive advantage these mutations might confer upon HSCs in the postchemotherapy setting.

Epidemiological data point to a correlation between *CHEK2* mutations in t-CH and the antecedent use of chemotherapeutic agents, particularly alkylating agents and platinum-based treatments, hinting at a selection process influenced by the genotoxic stress of such therapies (5). Notably, the paucity of *CHEK2* mutations within myeloid malignancies presents a question regarding the leukemogenic potential of *CHEK2*-mutated CH (6). The lack of these mutations in the context of myeloid transformation suggests that while they may be a marker of clonal selection in response to chemotherapy, their role in leukemogenesis might be limited or influenced by additional, as yet unidentified, factors.

Further complicating the narrative, germline *CHEK2* mutations have been implicated in predisposition to CH and myeloid malignancies (80, 81), indicating that the *CHEK2* mutation’s role in leukemogenesis is multifaceted and may be context dependent (82–84). Consistent with the observation that the mutations acquired in DDR genes are mutually exclusive, *CHEK2 germline mutation carriers do not acquire PPM1D* mutation. Instead, clonal expansion in *CHEK2* germline mutation carriers is accompanied by biallelic inactivation of *CHEK2* (75). Since *CHEK2* germline mutation carriers are at a higher risk of developing CH (82–84), the entire HSC pool may undergo accelerated acquisition of additional mutations, such as *CHEK2* biallelic or *DNMT3A* mutations, that drive transformation. Given the established role of CHEK2 in DNA repair and the maintenance of genomic integrity, the implications of its mutation in the postchemotherapy bone marrow HSCs demand further exploration to elucidate the potential for malignant transformation and to clarify its impact on the fitness of HSCs.

### Additional t-CH genes.

In addition to the most common t-CH genes discussed above, a variety of other genes are rarely found to be mutated in the general population, but appear in some studies in the context of exposure to cytotoxic agents. For example, mutations in *SRCAP*, which encodes a chromatin remodeler, have been reported after CAR-T cell therapy, bone marrow transplantation, and chemotherapy (7, 85). While these mutations can be found in both the general population and after chemotherapy, the mutations are significantly more frequent in patients previously treated with chemotherapy (70, 85). The VAF of *SRCAP* mutations can be as high as 8%, although no associated malignancies have been reported thus far (85). Experimental models showed the selective expansion of *SRCAP*-mutated cells under doxorubicin treatment and after stem cell transplant (85). Interestingly, *SRCAP*-mutated HSCs showed lymphoid bias, potentially implicating SRCAP’s role in lymphoid malignancies.

Mutations in the gene encoding *ATM*, one of the critical sensors of DNA damage (Figure 2), are reported in numerous studies, albeit at low frequency. Systematic studies of the role of these mutations have yet to be conducted. Still, given ATM’s importance in regulating p53, it can be hypothesized that mutations in ATM may reduce the activation of the CHEK2/p53 pathway in response to DNA damage, thereby partially phenocopying mutations in *TP53* and *PPM1D*. ATM may also regulate CH through its role in telomere maintenance, as variants in the telomerase reverse transcriptase (*TERT*) are associated with CH (63, 83, 84) and *ATM* loss in ataxia telangiectasia leads to telomere shortening (86).

Several other understudied genes appear in multiple studies, but their function and roles must be better understood. For example, *CUX1* mutations are found in t-CH (40, 70), myeloid malignancies (87), and other solid tumors (8). CUX1 is a nonclustered homeobox-transcription factor that plays a role in epigenetic regulation of DDR (88). HSCs deficient in CUX1 exhibit a selective advantage under alkylating chemotherapy compared with wild-type HSCs, ultimately contributing to the development of t-MNs in murine models (88). Moreover, *BAX*-mutated CH has been shown to arise after the treatment with a BCL-2 inhibitor, venetoclax, in patients with chronic lymphocytic leukemia (CLL) (89), strongly reflecting the evolutionary dynamics in response to targeting antiapoptotic proteins. However, the clinical implications of these *BAX*-mutated CH have not been elucidated.

One interesting question is, to what extent do specific mutations confer an advantage in the context of specific types of chemotherapy or particular drugs? This mutant-drug relationship is exemplified in the setting of *TP53* mutations, as discussed above (e.g., lenalidomide versus pomalidomide). Additionally, *PPM1D* mutations are more common in patients pretreated with platinum agents compared with other drug classes, a finding supported by in vitro studies (6). Similarly, in vitro studies on *SRCAP* mutants suggest selective expansion in the context of doxorubicin treatment (85). Accumulating this knowledge will likely contribute to clinical decision making regarding which chemotherapies to avoid in high-risk circumstances. However, the extent to which these biases are replicated in actual patients needs to be verified with actual patient cohorts.

### Structural variations in t-CH.

Structural variations (SVs), such as chromosome copy number alterations (CNAs) or chromosome rearrangements, have also been identified in CH and are often referred to as mosaic chromosomal alterations (mCAs) (90, 91). While the landscape of mCAs has been well characterized in dn-CH, their prevalence and characteristics in the context of t-CH remain poorly studied. Gao et al. analyzed 32,442 cancer patients in the MSK-IMPACT cohort and jointly called CH with gene mutations and mCAs. Due to the low-density SNP coverage, mCA was only detected in 346 patients (1%). Nonetheless, the study revealed that patients having t-CH in a form of both gene mutations and mCAs have the highest risk of developing secondary leukemia (91). In addition to mCAs, gene rearrangements constitute an important aspect of t-CH. For instance, the rearrangement of the *KMT2A* gene has been linked to the development of t-MNs following treatment with topoisomerase II inhibitors. However, due to the technical difficulties associated with screening for gene rearrangements with high sensitivity, the frequency of these rearrangements in t-CH remains unclear. Moreover, the functional characterization of mCAs and their role in clonal expansion is not well understood, partly due to the lack of appropriate models for studying mCAs. Thus, a systematic characterization of SVs in patients treated with chemotherapy is warranted.

## The leukemic potential of t-CH

Comprehensive studies into the leukemogenic potential of t-CH have been scarce, and it has yet to be discovered whether the patterns of progression from CH to leukemia observed in the de novo setting apply to the posttherapy landscape. In the general population, predictive models for CH transformation into hematologic malignancy have been developed (CH risk score [CHRS] or MN-predict), with varying levels of risk attributed to different mutations (65, 92). *TP53* mutations, while anecdotally included in these models based on associative studies, did not initially present as a high-risk factor (65). However, the presence of *TP53* mutations is significantly elevated in the context of t-MN (6, 8), which might suggest a heightened leukemic potential when these mutations are stressed under chemotherapy. This hypothesis is supported by data indicating that chemotherapy and radiation therapy could potentially select *TP53*-mutant clones, thereby accelerating their pathogenic evolution.

The role of *PPM1D* mutations in leukemogenesis still needs to be clarified. Although these mutations confer a survival advantage under the selective pressure of chemotherapy, their direct contribution to the development of leukemia has not been conclusively demonstrated. The selective advantage conferred by chemotherapy and the unclear path to malignancy underscore the complexity of the relationship between t-CH and leukemic transformation.

## The interplay between t-CH and cancer outcomes

The relationship between t-CH and cancer outcomes is an area of active investigation, particularly in light of evidence suggesting that CH can provoke an aberrant systemic inflammatory response with potential implications for cancer progression and response to therapy. Additionally, t-CH can serve as a precursor for the development of t-MNs, which may adversely affect the survival of cancer survivors. In the unselected cancer patient cohort, t-CH has been linked with diminished overall survival and an increased risk of secondary hematologic malignancies (4). While t-CH is clearly linked with an elevated risk of t-MN development, it is important to note that t-MN remains a relatively rare complication, and its impact on survival at the population level is still limited. Therefore, the negative impact of t-CH on cancer patient survival may also be driven by other factors such as cardiovascular mortality and others (4).

Additionally, the impact of t-CH on cancer outcomes appears to be context dependent, influenced by the specific types of cancer and the treatments administered. Several investigations have been conducted to elucidate the associations between t-CH and outcomes in various cancers, including colon (36) and lung cancers (22, 37). These studies have yielded mixed findings, indicating that the prognostic significance of t-CH may not be universally applicable across different cancer types. For instance, Arends et al. identified a paradoxical association where t-CH correlated with improved survival outcomes in metastatic colon cancer patients receiving FOLFIRI-based regimens (36). In contrast, another study found that t-CH had no discernible effect on the overall survival of gastrointestinal cancer patients, including colorectal cancer patients treated with chemotherapy and/or immune checkpoint inhibitors (29). This suggests that the implications of t-CH are complex and may be heavily context dependent.

The potential influence of CH on the efficacy of cancer immunotherapy presents another intriguing aspect of CH’s impact on cancer management. In a mouse model, *Tet2* deletion in myeloid cells suppressed melanoma growth by activating tumor-associated macrophages into a proinflammatory state (93). Additionally, the deletion of *Dnmt3a* in CD8 T cells led to the retention of effector function and proliferative capacity and prevented T cell exhaustion (94). These data suggest that the presence of CH cells in the tumor microenvironment could potentially alter antitumor immunity and modify the treatment efficacy of cancer immunotherapy, such as immune checkpoint inhibitors or tumor-infiltrating lymphocyte (TIL) therapies.

The diversity in outcomes highlights the complexity of t-CH as a clinical entity and its multifaceted impact on cancer prognosis. It underscores the necessity for a more granular understanding of how t-CH interacts with the underlying cancer biology and the effects of various treatment modalities. Further research is essential to delineate the precise mechanisms through which t-CH influences cancer progression and determine whether its detection can effectively guide treatment strategies.

## Systemic implications of t-CH beyond oncology

t-CH has garnered attention not only for its implications in cancer outcomes, but also for its potential systemic effects. Atherothrombotic cardiovascular disease represents the most established association for dn-CH, particularly in CH with mutations in genes such as *DNMT3A* and *TET2* (95, 96). However, the extrapolation of these findings to t-CH remains speculative, as systematic studies specific to t-CH’s impact on cardiovascular disease have not yet been conducted.

Animal studies have provided some evidence that *TP53* and *PPM1D* mutations can lead to aberrant cardiac responses (97–99). These findings resonate with observations in broader patient cohorts, where *TP53* mutations have been associated with an augmented risk of atherosclerotic cardiovascular disease (99). These associations suggest a possible amplification of cardiovascular risk factors in patients with t-CH, although the clinical relevance and mechanisms remain to be fully elucidated.

**Figure 3:**
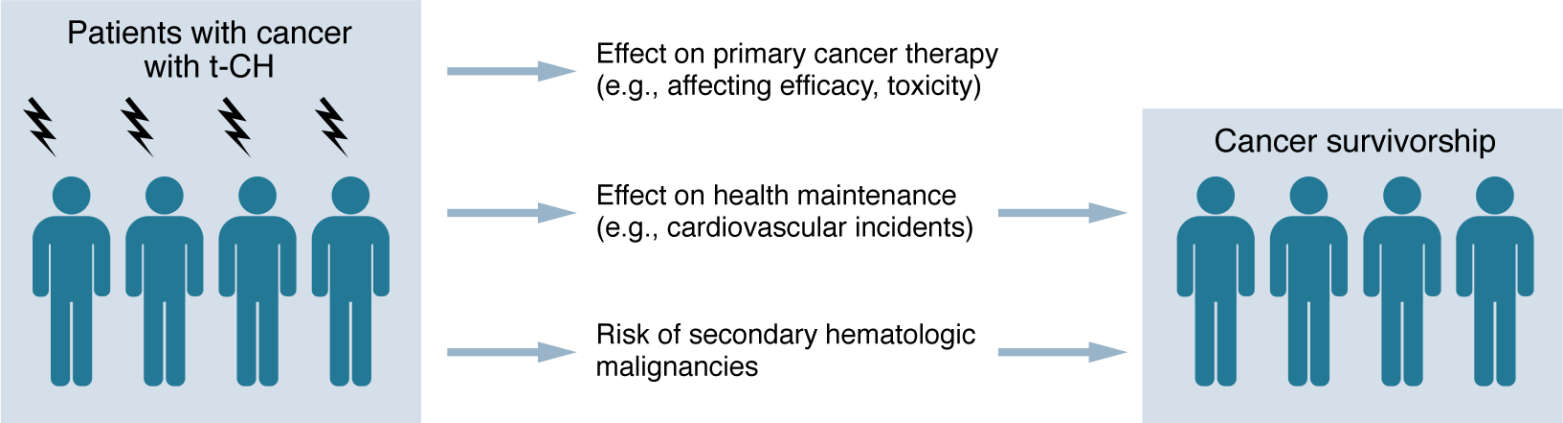
Clinical implications of t-CH in patients with cancer and survivorship.

Beyond cardiovascular disease, CH has been linked to various other inflammatory and degenerative conditions, such as osteoporosis (100), Alzheimer’s disease (101), chronic obstructive pulmonary disease (COPD) (102), and others. However, the ramifications of these associations within the context of t-CH and the oncology patient population need to be clarified. The outcomes for cancer patients are predominantly influenced by the cancer itself, which might overshadow or dilute the prognostic impact of concurrent CH. Nevertheless, as the number of cancer survivors continues to increase, t-CH and its potential impact on cardiovascular events, secondary hematologic malignancies, and other conditions are becoming increasingly important for cancer survivorship. It is not coincidental that a growing number of CH clinics are being established primarily within large cancer centers (2). To provide comprehensive survivorship care for patients with t-CH after cancer therapy, a multidisciplinary approach involving oncologists, cardiologists, and other specialists is essential (Figure 3) (2).

Future research is necessary to dissect the myriad ways t-CH might contribute to systemic disease, potentially affecting both the progression of preexisting conditions and the emergence of new pathologies. Understanding these relationships is crucial for developing a holistic approach to managing patients with t-CH, ensuring that both oncologic and nononcologic aspects of health are addressed.

## Concluding remarks

t-CH has emerged as a distinct clinical entity akin to t-MNs, which are recognized by the World Health Organization and other medical classifications for their unique clinical and pathological characteristics (103). The mutational landscape that defines t-CH is notably distinct from that of dn-CH, conferring specific risks and bearing particular clinical significance. These differences underscore the importance of acknowledging t-CH as a separate category within the spectrum of hematopoietic clonal disorders.

The clinical ramifications of t-CH are multifaceted, shaped not only by the intrinsic biological consequences of somatic mutations within HSCs, but also by the intricate interplay with chemotherapeutic and other therapeutic interventions. The relationship between t-CH and treatment outcomes is context dependent, with both beneficial and deleterious effects being reported. This highlights the complexity of these interactions and the need for flexible clinical strategies.

In the real-world clinical setting, where CH is more frequently detected in cancer patients than the general population, it is critical to intensify research efforts focused on t-CH. An increased understanding of t-CH could lead to significant advancements in the prognostication and management of patients who have undergone cancer therapy, ultimately improving their overall care and outcomes.

## Author contributions

KT, DN, and MG wrote the manuscript and approved the final version.

## Figures and Tables

**Table 3:**
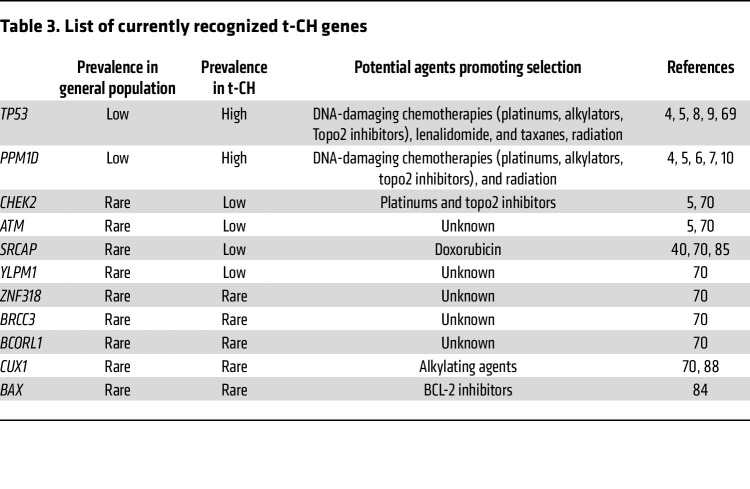
List of currently recognized t-CH genes

**Table 1:**
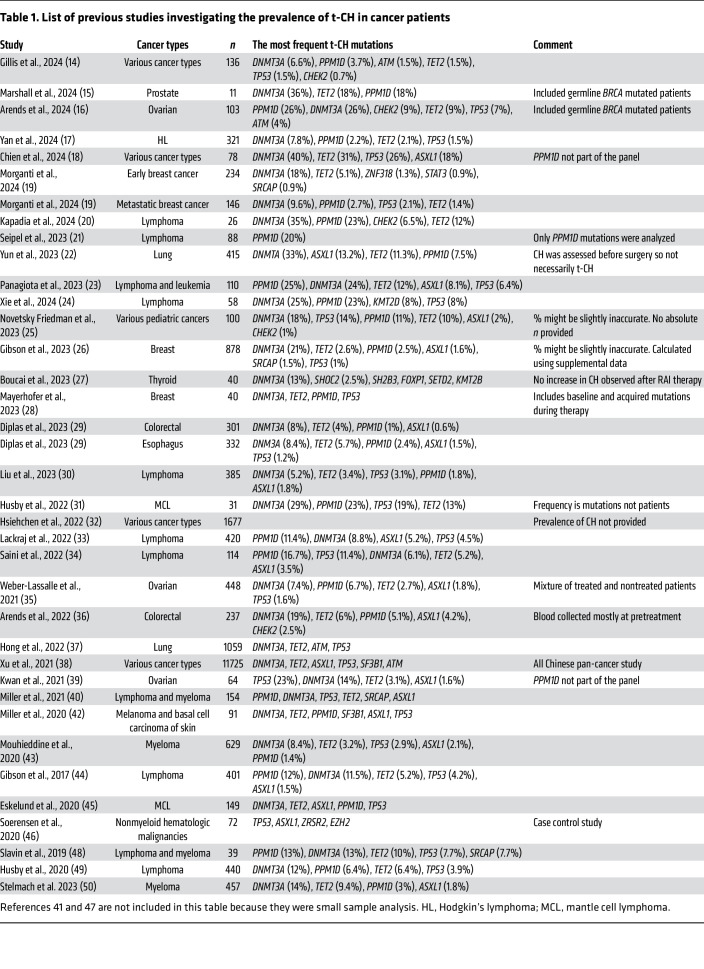
List of previous studies investigating the prevalence of t-CH in cancer patients

**Table 2:**
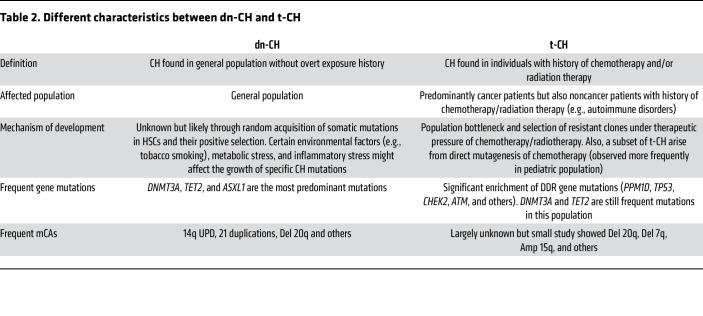
Different characteristics between dn-CH and t-CH

## Version 1 10/01/2024

Electronic publication
